# Different wheat cultivars exhibit variable responses to inoculation with arbuscular mycorrhizal fungi from organic and conventional farms

**DOI:** 10.1371/journal.pone.0233878

**Published:** 2020-05-29

**Authors:** David García de León, Tanel Vahter, Martin Zobel, Mati Koppel, Liina Edesi, John Davison, Saleh Al-Quraishy, Wael N. Hozzein, Mari Moora, Jane Oja, Martti Vasar, Maarja Öpik

**Affiliations:** 1 Department of Life Sciences, Technological Science Campus, University of Alcalá, Alcalá de Henares, Spain; 2 Institute of Ecology and Earth Sciences, University of Tartu, Tartu, Estonia; 3 Zoology Department, College of Science, King Saud University, Riyadh, Saudi Arabia; 4 Department of Botany, University of Tartu, Tartu, Estonia; 5 Estonian Crop Research Institute, Jõgeva, Estonia; 6 Botany and Microbiology Department, Faculty of Science, Beni-Suef University, Beni-Suef, Egypt; University of Nebraska-Lincoln, UNITED STATES

## Abstract

The present study aimed to investigate the effects of arbuscular mycorrhizal (AM) fungal communities originating from organic and conventional agriculture on wheat growth and yield. Six different spring wheat cultivars released in different years in north and central European countries were considered. We hypothesised that AM fungal inoculum collected from organic agricultural fields would elicit a greater positive growth response than inoculum collected from conventional agricultural fields; and that older cultivars, which were developed under conditions of low fertilizer input, would exhibit overall greater growth responses to the presence of AM fungi, compared with more recent cultivars, and that AM fungal inoculum from conventional fields might have the most beneficial effect on the growth and yield of recent cultivars. The results showed that the overall effects on the growth and yield of spring wheat grown with organic and conventional AM fungal inocula did not differ greatly. However, the inoculation growth response, showing the difference of the effects of organic and conventional inocula, varied between particular wheat cultivars. Inoculation growth response of the cultivar Pikker (released in 1959) was the most positive, while that of the cultivar Arabella (released in 2012) was the most negative. The use of AM fungal inoculum from organic fields resulted in slightly taller plant individuals. Pikker showed relatively higher yield and stronger growth when the organic AM fungal inoculum was used. Arabella exhibited relatively lower yield and weaker growth when the organic inoculum was used. Whether the positive response of Pikker to Estonian organic inoculation reflects adaptation to the locally occurring AM fungal community needs to be established by further studies of the communities of AM fungi colonizing wheat roots.

## Introduction

Soil organisms are integral components of ecosystems, and healthy soils that contain an active microbiome are critical for the sustainable production of food for the expanding global human population [1]. Nonetheless, soil microorganisms still receive relatively little recognition in agricultural management strategies [2].

Arbuscular mycorrhizal (AM) fungi (phylum Mucoromycota, subphylum Glomeromycotina) [3] represent one of the most ubiquitous groups of soil microorganisms, colonizing the roots of about 80% of terrestrial plant species [4]. AM fungi provide their host plants with nutrients (mainly P and N) and receive plant assimilated carbon in exchange [4]. In addition, AM fungi provide further benefits to plants such as alleviating moisture stress [5] and increasing plant resistance to pathogens [6]. There is also emerging evidence that AM fungi reduce leaching of nutrients from soil by enlarging the nutrient interception zone [7].

AM fungi play important roles in agroecosystems, including cereal cultivation. The evolutionary history and taxonomic distribution of AM interactions suggest that the progenitors of modern cereal crops inherited the capacity to interact with Glomeromycotinan fungi [8]. Wheat (*Triticum* spp.) is a major food crop that is widely grown around the world under diverse climatic conditions with a total of 756.7 million tons of wheat produced in 2018 [9]. It is classified as a non-mycorrhizal or mycorrhizal plant species depending on the cultivar [10]. AM fungi can enhance P nutrition and growth of wheat in experimental conditions [11,12], accordingly, suppression of AM fungi in the field can decrease the yield of wheat [13]. Tracking of radioactive P has shown that assemblages of indigenous AM fungi make a significant contribution to P uptake by wheat [14]. In addition, there have been several attempts to increase wheat nutrient uptake, growth and yield by applying extra AM fungal inoculum in the field. Meta-analyses have shown that AM fungal inoculation can increase aboveground biomass, grain yield, and P and N concentrations of wheat [15,16].

Wheat cultivars associate with different AM fungal communities in their roots [17] and may respond differently to AM fungal inoculation in terms of growth, root colonization and carbon for nutrient exchange [18–20]. Hetrick et al. [10] suggested that the age of a cultivar (i.e. its year of release) could be an important determinant of its inoculation response. Their study of 20 wheat cultivars under greenhouse conditions revealed that cultivars released before 1950 profited more consistently from AM fungal inoculation in terms of biomass, while the response of cultivars released after 1950 was more variable. They concluded that recent agricultural and plant breeding practices may have favoured the development of cultivars that effectively exploit highly fertilized systems with less reliance on mutualists. However, later studies have shown varying results. A meta-analysis [21] showed no evidence that new wheat genotypes have lost their ability to respond to mycorrhizal fungi. On the other hand, a recent meta-analysis by Zhang et al [16] showed that there is a tendency of newer wheat varieties showing decreased response to AM fungi in terms of yield.

Organic farming represents a type of agroecosystem where AM fungi may be more beneficial than in conventional farming, where inorganic fertilizers are provided [22]. Indeed, several studies have shown that soil AM fungal communities from organically managed farms differ in taxonomic composition in comparison with conventional farms [23–26]. However, the effects that AM fungal communities associated with organic and conventional farming have on the growth and yield of cereals are unclear. For instance, in an experiment with maize, Verbruggen et al. [27] failed to find a positive growth effect of AM fungal inoculum originating from organic farming. While Gottshall et al. [28] reported a positive effect of organic field inoculum on wheat growth.

Inoculation experiments with wheat have mostly used cultured AM fungal strains [18]. Cultured fungi are believed to exhibit specific traits compared with uncultured fungal taxa, which commonly colonize plant roots in the field alongside the cultured taxa [29]. Because of this, it would be highly desirable to study the response of wheat cultivars not only to cultured fungi, but also to the AM fungal communities naturally occurring in their agroecosystems. In particular, understanding the effects of AM fungal communities from the soil of organic and conventional farms would be of interest.

We aimed to study the effect of AM fungal communities, originating from organic and conventional farming on the growth and yield of wheat. We hypothesized that AM fungal inoculum collected from organic fields will result in a higher positive growth response than inoculum collected from conventional fields. We used six spring wheat cultivars in the experiment and hypothesized that older cultivars, which were developed under conditions of low fertilizer input, will exhibit stronger AM fungal growth responses compared with more recent cultivars. Similarly, we hypothesized that AM fungal inoculum from conventional fields might more effectively enhance the growth and yield of recent, compared with older, cultivars.

## Material and methods

A greenhouse experiment was performed at the Estonian Crop Research Institute, Jõgeva, during August-December 2017.

### Wheat cultivars

Six spring wheat cultivars were used in the experiment: Diamant, Pikker, Tähti, Runar, Arabella and Sorbas (Table 1). The older cultivars, Diamant—released in 1929 and Pikker—released in 1959, were developed through selective breeding at a time when the use of mineral fertilizers was minimal and pesticides were not used. In contrast, the most recent cultivars, Arabella—released in 2012 and Sorbas—released in 2016, were developed under conditions of intensive use of fertilizer and pesticide application.

**Table 1:** Wheat cultivars used in the trial.

Cultivar	Year of release	Country of origin
Diamant	1929	Sweden
Pikker	1959	Estonia
Tähti	1972	Finland
Runar	1972	Norway
Arabella	2012	Poland
Sorbas	2016	Germany

### Origin of soil inocula

Soil inocula were collected from adjacent organic and conventional fields in the Nissi—Märjamaa region of North-Estonia in April 2017. It was aimed to get soil from sites with as similar edaphic conditions as possible. Selected fields were on Calcaric—Mollic Gleysols with sandy loam texture. Soil inocula were collected from two sites with organic fields and from two sites with conventional fields. From each site, ten randomly chosen locations were used for soil collection. Local heterogeneity was not being tested so all organic and all conventional soil samples were pooled. Spring barley was cultivated in the previous growing season in all fields used for inoculum collection.

### Experimental set up

The effect of soil inoculum origin (organic versus conventional fields) was explored on the growth of six wheat cultivars. Seeds were surface sterilized prior to sowing: 5 minutes in 70% ethanol, followed with 15 minutes sterilization in 5% sodium hypochlorite and rinsed three times with distilled water. Three seeds were sown per each plastic pot (19 × 14 cm, depth x diameter) in August 2017. One seedling was retained per pot after 2 weeks of growth. The growth substrate was a mixture of the two natural soils (i.e., from conventional and organic fields), with one soil being sterilized by gamma irradiation (20 h at 1 kGy) and the other soil serving as the live inoculum. Thus, two growth substrates were prepared: organic (a mixture of gamma sterilized conventional soil and intact organic soil) and conventional (a mixture of gamma sterilized organic soil and intact conventional soil). In order to restore the microbial community of sterilized soil except for AM fungal community, all pots then received 40 ml of filtered (pore size 50 μm) mixed soil inoculum wash to correct for possible differences in soil bacterial and non-AM fungal communities [30]. This approach is widely used because most AM fungal spores do not penetrate the filter with 50 μm pore size [31–35]. Root colonization was checked in sterile controls. There was almost no colonization (average colonization ± standard deviation, 0.5 ± 1.3%), which proofed that microbial wash did not introduce AM fungi.

Each combination of soil inoculum treatment (altogether two) and spring wheat cultivar (altogether six) was replicated 10 times, producing a total of 120 pots. The experiment was conducted in a greenhouse with a day length of 16 h at the temperature regime 20+/-2°C day/ 12+/-2°C night. The plants were watered when necessary. Fertilizers and pesticides were not used. The experiment was harvested in December 2017, after 16 weeks of growth.

### Trait measurements

At the end of the experiment, the height of each plant individual was measured. Thereafter, grains, shoots and roots were harvested separately, dried at 70°C for 48 h and weighed. The fine roots of harvested plants were stained with trypan blue and the percentage of mycorrhizal fungal colonization was estimated using the magnified grid line intersection method [36], as per Uibopuu et al [33]. Specifically, the root colonization rate was computed as the sum of the area colonized by hyphae, vesicles, arbuscules, dark septate hyphae, and spores. The fine roots of three randomly harvested plants within each treatment combination were retained for further molecular analysis. Root colonization is summarized in S1 Table.

### Soil chemical analyses

Concentration of soil P, potassium (K), calcium (Ca), magnesium (Mg), copper (Cu), manganese (Mn), boron (B), N and pH were measured as in Garcia de Leon et al. [37]. The Mehlich III procedure was used to determine the soil available nutrients: P, K, Ca, Mg, Cu and Mn after extracted by reaction with acetic acid and fluoride compounds [38]. Mehlich III provides estimates of P that are well correlated with alternative techniques. Soil B was extracted and determined according to the method of Berger and Truog [39]. Soil total N was measured using the Kjeldahl method according to ISO 11261:2002 [40]. Soil pH was measured in KCl following the standard method in Sumners [41]. Chemical analyses were performed at the laboratory of the Agricultural Research Centre in Saku, Harjumaa, Estonia.

### Molecular analyses

Molecular analyses were used to identify the effects of experimental treatments on AM fungal communities in the roots of wheat plant individuals. DNA was extracted from 70 mg dried roots using a DNeasy Plant Mini Kit [42]. AM fungal sequences were amplified from root DNA extracts using AM fungal specific primers for the small-subunit (SSU) ribosomal RNA gene: WANDA [43] and AML2 [44]. A first PCR was conducted with amplicon specific primers linked to Illumina Nextera XT sequencing adapters (Illumina forward primer adaptor: 5′-TCGTCGGCAGCGTCAGATGTGTATAAGAGACAG-3′; Illumina reverse primer adaptor: 5′-GTCTCGTGGGCTCGGAGATGTGTATAAGAGACAG-3′). The reaction mix contained 12.5 μl KAPA HiFi Hotstart PCR mix, 1.0 μl of each 10 μM primer, 6 μl of template DNA and MQ water to reach a total reaction volume of 25 μl. This relatively high volume is due to the low amount of AM fungal DNA usually found and the fact that DNA concentrations were not normalised in the PCR step in order that the read count could be used to represent taxon abundance. In case of samples with very low AM fungal abundance, this amount will yield sufficient PCR product for sequencing and to avoid false negatives.

The PCR was performed under the following cycling conditions: 95°C for 3 min, 40 cycles of 95°C for 30 s, 55°C for 30 s, 72°C for 30 s followed by 72°C for 5 min. A nested PCR was performed with Nextera XT index-adapters. The reaction mix contained 15 μl KAPA HiFi Hotstart PCR mix, 5 μl of Nextera XT index 1 Primer (N7xxx), 5 μl of Nextera XT index 2 Primer (E5xxx), 5 μl of DNA (10 ng μl^−1^) and MQ water to reach a total reaction volume of 30 μl. The PCR was performed under the following cycling conditions: 95°C for 3 min, seven cycles of 95°C for 30 s, 55°C for 30 s, 72°C for 30 s followed by 72°C for 5 min. After the second PCR, the PCR products were purified with Agencourt AMPure XP beads. The resulting mix was sequenced on the Illumina MiSeq platform using a 2 × 300 bp paired-end sequencing approach at Asper Biogene (Tartu, Estonia).

### Bioinformatic analyses

Illumina 2x300 bp paired-end raw reads (2 x 1 913 301 reads in total) were demultiplexed into samples and cleaned using a series of bioinformatic steps [45]. Reads were demultiplexed by checking double barcodes, allowing one mismatch for both reads. Reads were retained if they carried the correct primer sequences (WANDA and AML2; allowing one mismatch for each) and had an average quality of at least 30 (after removal of primer and barcode sequences), and orphan reads were removed (leaving 2 x 1 131 874 cleaned reads). Paired-end reads were combined with FLASh v1.2.10 [46] using the default parameters—overlap at least 10 bp and overlap identity at least 75%—to leave 1 125 036 combined reads (99.4% success rate). Putative chimeric sequences (6 568; 0.58% of cleaned reads) were identified and removed using vsearch v2.14.1 [47] with the default parameters in reference database mode against MaarjAM database (status June 2019 [48]). Cleaned and chimera free sequences were assigned to virtual taxa (VT) from the MaarjAM database using BLAST+ v2.5.0 [49]. With the blastn algorithm, BLAST+ hits were filtered based on best hit using 97% identity and 95% alignment. Representative sequences of each virtual taxon were submitted to EMBL database (accession number ERP119882).

### Statistical analyses

Plant height, root weight, shoot weight, grain weight, proportional grain weight, and percentage root colonization were calculated. The proportional grain weight was the ratio between grain weight and the summed of all other fractions. Proportional grain weight was a coarse proxy of crop yield, because grain is the main wheat product. We calculated the Inoculation Responsiveness (IR) on standardized data following Gottshall et al. [28] by subtracting the average plant trait value with conventional inoculum from the average plant trait value with organic inoculum. Differences among the Inoculation Responsiveness (IR) of cultivars could not be statistically tested due to the fact that this proxy was based on average values (i.e. there was a lack of within-cultivar variability per plant trait). S1 Fig compares the Inoculation Responsiveness with Mycorrhizal Responsiveness (MR), where the minuend is the average plant trait value with organic field inoculum, or alternatively conventional field inoculum, and the subtrahend is the average plant trait value with sterile soil.

The assessment of inoculum source on plant traits effects, AM fungal richness and read abundance, cultivar and their interaction were with linear models. Because AM fungal sample taxon accumulation curves (S2 Fig) showed no relationship between the number of sequences obtained from a sample and the taxon richness of the sample, we performed fungal richness analyses on unrarefied richness data. The significance of terms was measured with F tests and Tukey post hoc tests in R [50].

To compare AM fungal communities between treatments and varieties, Bray-Curtis dissimilarity of relative abundances was the measure of the distance between communities. Non-metric multidimensional scaling (NMDS) using the *metaMDS* function in the *vegan R package* [51] was used to visualize the separation of communities. To test for differences in community composition, PERMANOVA with 999 permutations was used (function *adonis* in the *vegan* R package).

## Results

### Average wheat response to mycorrhizal inoculation

**Fig 1:**
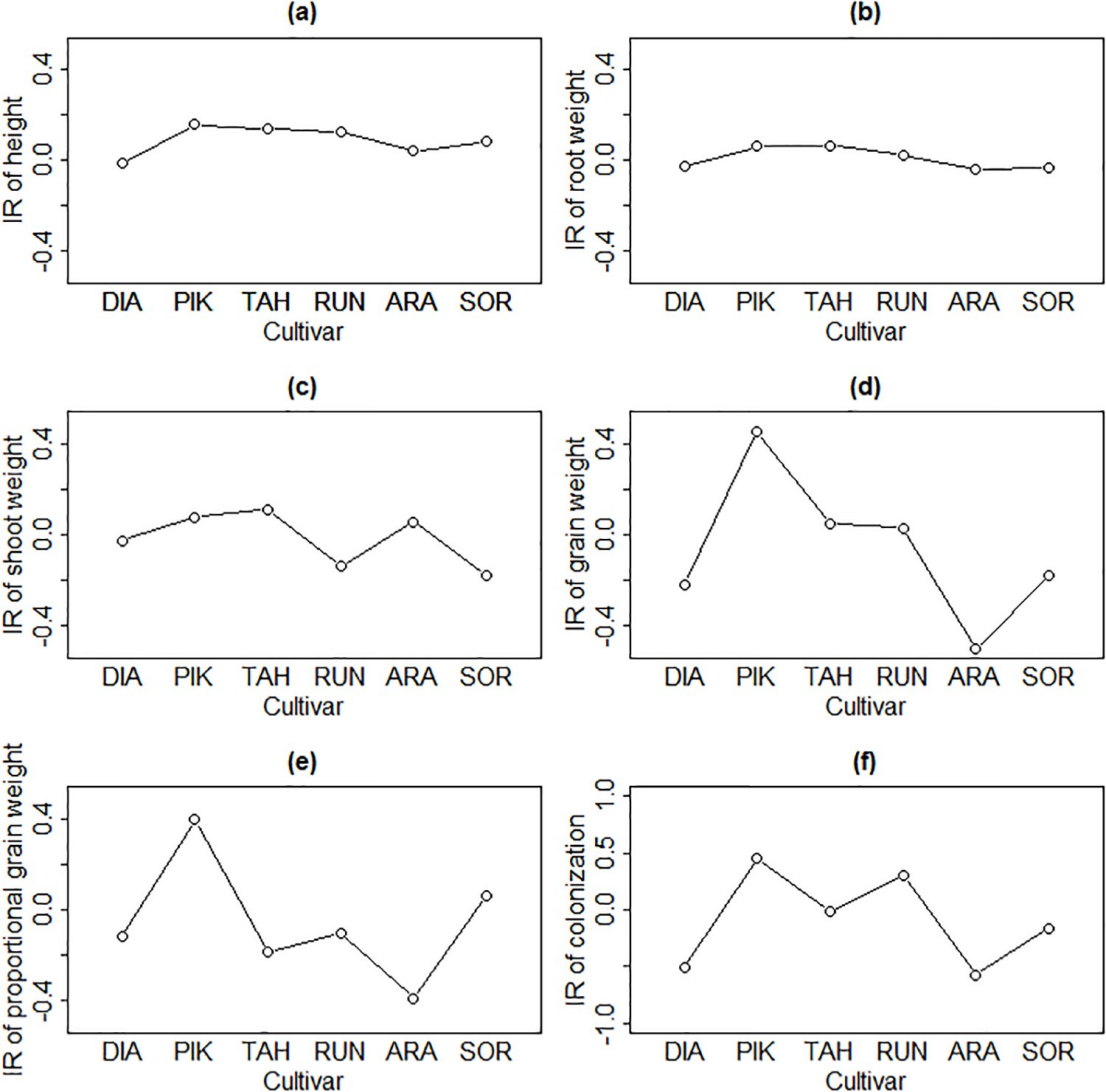
Average Inoculation Responsiveness (IR) of wheat cultivar traits, showing the difference of the effects of organic and conventional inocula on: (a) plant height, (b) root weight, (c) shoot weight, (d) grain weight, (e) proportional grain weight, and (f) root colonization. Higher value of IR exhibits stronger positive effect of organic inoculum. DIA: Diamant: 1929, PIK: Pikker 1959, TAH: Tähti 1972, RUN: Runar 1972, ARA: Arabella 2012, SOR: Sorbas 2016. Mycorrhizal responsiveness was calculated on standardized data as: ((average plant trait value growing with organic field inoculum—average plant trait value growing with conventional field inoculum). Differences among the inoculation responsiveness of cultivars could not be statistically tested due to the fact that this proxy was based on average values (i.e. there was a lack of within-cultivar variability per plant trait).

Table 2 summarizes soil nutrients and pH per plot. The inoculation response, i.e. the growth effect of mycorrhizal fungal inoculation under organic inoculum compared to conventional inoculum varied among wheat cultivars. Cultivar Pikker 1959 recorded the most positive mycorrhizal response of the plant height, the weight of roots, shoot weight, and grain the proportional grain weight (Fig 1, S2 Fig). By contrast, cultivar Arabella 2012 was the most negatively affected by mycorrhizal fungal inoculation for most plant traits.

**Table 2:** The properties of soil mixtures collected from organically and conventionally managed arable fields.

	P	K	Ca	Mg	Cu	Mn	B	N	pH_KCl_
	mg kg^-1^							%	
Organic	52	199	9656	218	2.6	24	2.73	0.46	7.1
Conventional	20	275	7454	314	5.3	19	2.01	0.46	7.0

### Inoculum effect

**Fig 2:**
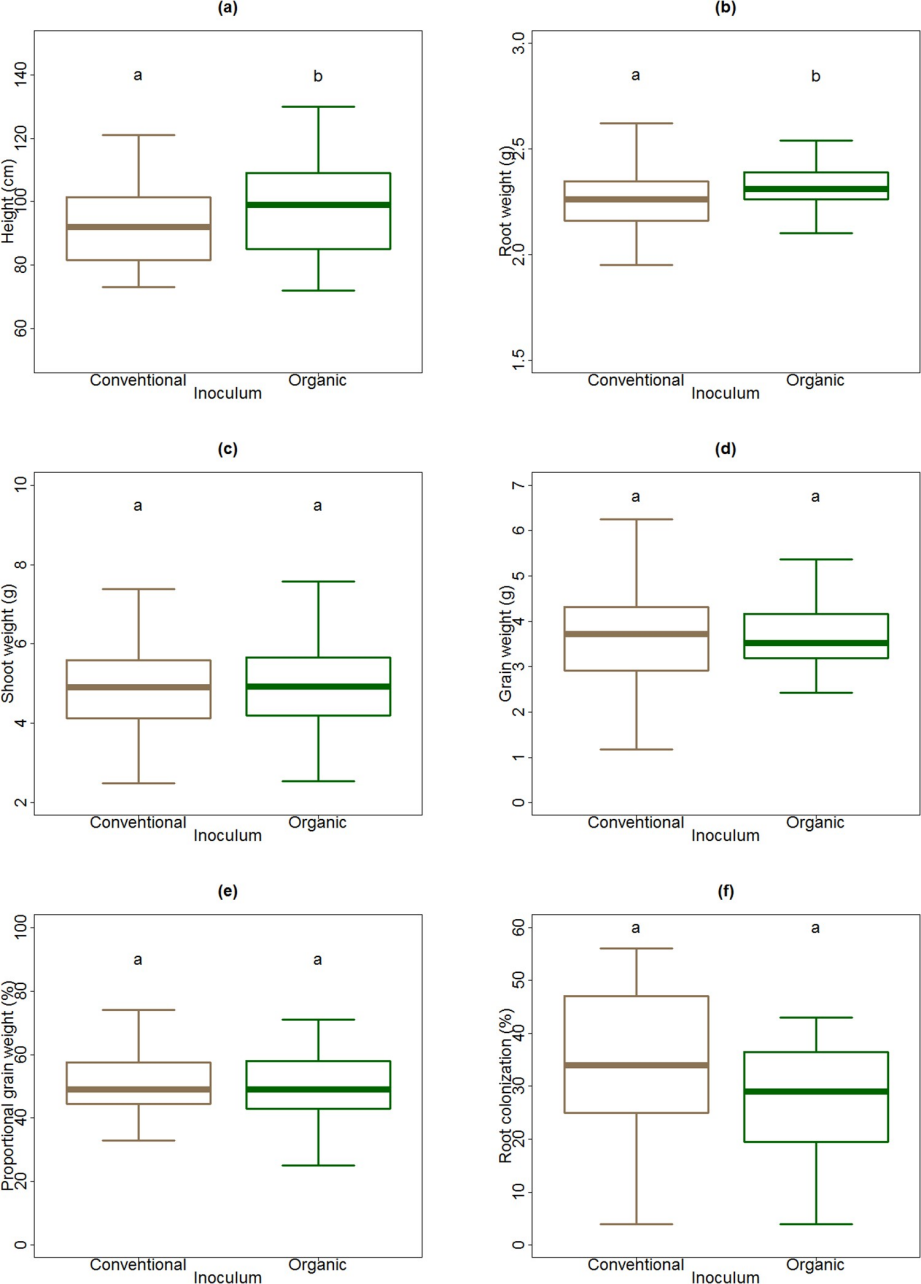
Effect of inoculum type (soil from conventionally or organically managed field) on plant traits: Height (a), root weight (b), shoot weight (c), grain weight (d), proportional grain weight (e), and root colonization (f). Thick lines represent medians; boxes indicate interquartile ranges; and whiskers show maximum and minimum values per sample.

Plants with organic field inoculum grew taller (F_1,104_ = 21.8, *P* < 0.01, Fig 2a), and produced larger roots (F_1,104_ = 4.0, *P* = 0.05, Fig 2b) compared with those grown with conventional field inoculum. The type of inoculum did not have a significant effect on the shoot weight (F_1,104_ < 0.01, *P* = 0.98, Fig 2c), grain weight (F_1,104_ = 1.7, *P* = 0.19, Fig 2d) or the proportional grain weight (F_1,104_ = 3.19, P = 0.08, Fig 2e). Root colonization trended to be smaller in organic field inoculum (average colonization ± standard deviation, 28.5± 13.5%), than in conventional field inoculum (average colonization ± standard deviation, 34.2± 14.8%). However, this difference was not significant (F_1,40_ = 1.46, *P* = 0.23, Fig 2f).

### Cultivar effect

**Fig 3:**
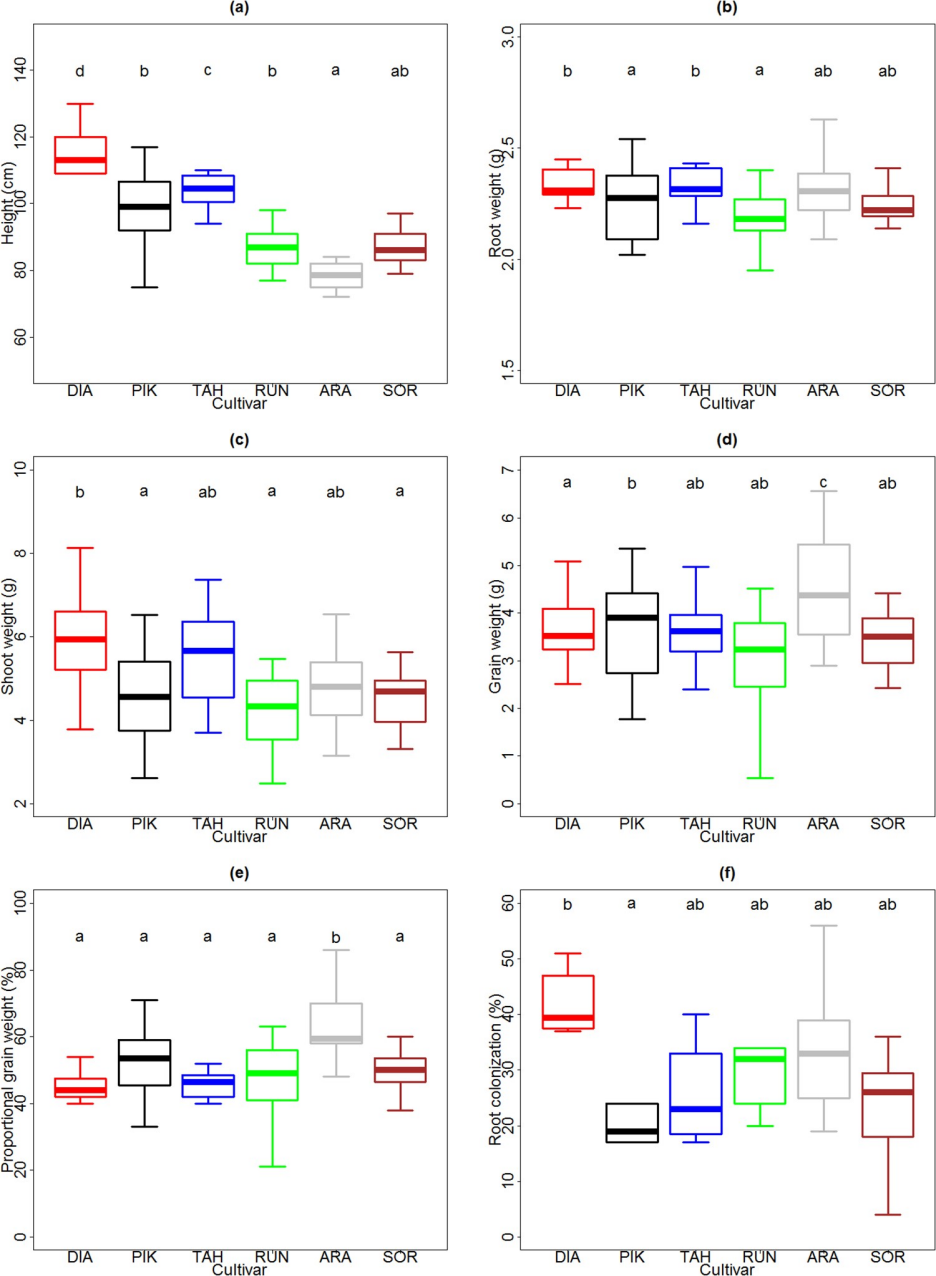
Differences between wheat cultivars in terms of plant traits: Height (a), root weight (b), shoot weight (c), grain weight (d), and proportional grain weight (e), and root colonization (f). f probably exaggerates the impression of differences, given that many bars represent 1–3 points. Thick lines represent medians; boxes indicate interquartile ranges; and whiskers show maximum and minimum values per sample. DIA: Diamant: 1929, PIK: Pikker 1959, TAH: Tähti 1972, RUN: Runar 1972, ARA: Arabella 2012, SOR: Sorbas 2016.

There were significant differences among the six cultivars of spring wheat with respect to all measured plant traits. There was a trend for cultivars to become shorter over time (i.e. from earlier to later release date; F_5,104_ = 80.3, *P* < 0.01, Fig 3a). The tallest cultivar was Diamant 1929 (average height ± standard deviation, 115.6 ± 6.7 cm), followed by Tähti 1972 (104.0 ± 7.1 cm), Pikker 1959 (100.1 ± 9.8 cm), Runar 1972 (89.0 ± 6.3 cm), Sorbas 2016 (87.8 ± 5.7 cm) and Arabella 2012 (77.4 ± 6.1 cm).

Plant height (F = 61.69, p <0.01, Fig 3a), root weight (F_5,104_ = 4.83, *P* < 0.01, Fig 3b) and shoot weight (F_5,104_ = 8.87, *P* < 0.01, Fig 3c) decreased from earlier to later cultivar’s year of release. On the other hand, grain weight increased (F_5,104_ = 7.33, *P* < 0.01, Fig 3d) and proportional grain weight rose (F_5,104_ = 14.97, *P* < 0.01, Fig 3e). In brief, new cultivars had shorter and smaller shoots. The percentage of root AM fungal colonization in cultivar Diamant 1929 was higher than in Pikker 1959 (F_5,40_ = 11.42, *P* = 0.04, Fig 3f).

### Interaction of inoculum and cultivar

**Fig 4:**
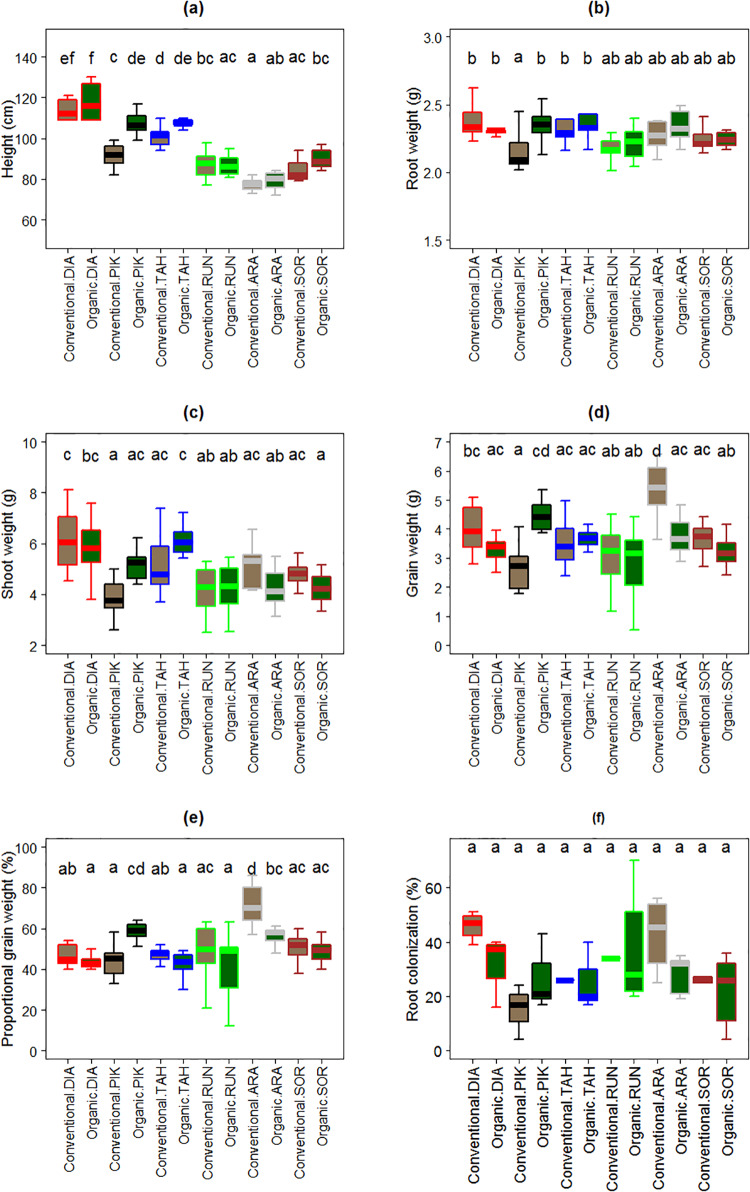
Effect of the interaction between inoculum type (soil from conventionally or organically managed field) and wheat cultivar on plant traits: Height (a), root weight (b), shoot weight (c), grain weight (d), proportional grain weight (e), and root colonization (f). Fig 4f probably exaggerates the impression of differences, given that many bars represent 1–3 points. Thick lines represent medians; boxes indicate interquartile ranges; and whiskers show maximum and minimum values per sample. DIA: Diamant: 1929, PIK: Pikker 1959, TAH: Tähti 1972, RUN: Runar 1972, ARA: Arabella 2012, SOR: Sorbas 2016.

All cultivars except Runar 1972 grew taller with organic than conventional field inoculum (F_5,104_ = 3.64, *P* < 0.01, Fig 4a), whereas no changes were observed for root weight (F_5,104_ = 1.83, *P* = 0.11, Fig 4b). Organic field inoculum increased the shoot weight of Pikker 1959 and Tähti 1972, while it decreased the shoot weight of all other four cultivars (F_5,104_ = 3.53, *P* < 0.01, Fig 4c). Although, interactions between inoculum type and cultivar were significant for grain weight (F_5,104_ = 9.36, *P* < 0.01, Fig 4d) and proportional grain weight (F_5,104_ = 7.33, *P* < 0.01, Fig 4e), there were no clear patterns observed in relation to the age of the cultivar.

Arabella 2012 –the shortest and one of the most recently released wheat cultivars under study- exhibited the highest grain weight (> 6 g per grain) and proportional grain weight (> 80%) under conventional field inoculum conditions. By contrast, Pikker 1959 –one of the tallest and earliest released varieties traditionally cropped in Estonia—displayed the largest increase in grain weight, and proportional grain weight when grown with organic field inoculum. With the exception of cultivar Pikker 1959, root AM fungal colonization trended to be higher in the presence of conventional field inoculum. Such a trend was not significant (F_5,40_ = 5.11, *P* = 0.40, Fig 4f).

### AM fungal community

Following molecular analyses and bioinformatics, 717,839 cleaned and quality filtered sequences were obtained. These yielded 85,064 hits against AM fungal VT, resulting in the identification of 24 AM fungal virtual taxa in the dataset (54,461 hits yielding 19 VT with conventional inoculum and 30,598 hits yielding 21 VT with organic inoculum, S2 Table). The five most dominant VT in the dataset made up 96% of all sequences and were members of the genera *Glomus* (VT388–40%), *Rhizoglomus irregulare* species complex (VT113–30%, VT115–21%, VT114–4%) and *Funneliformis* (VT67 related to *Funneliformis coronatum*– 1.5%).

**Fig 5:**
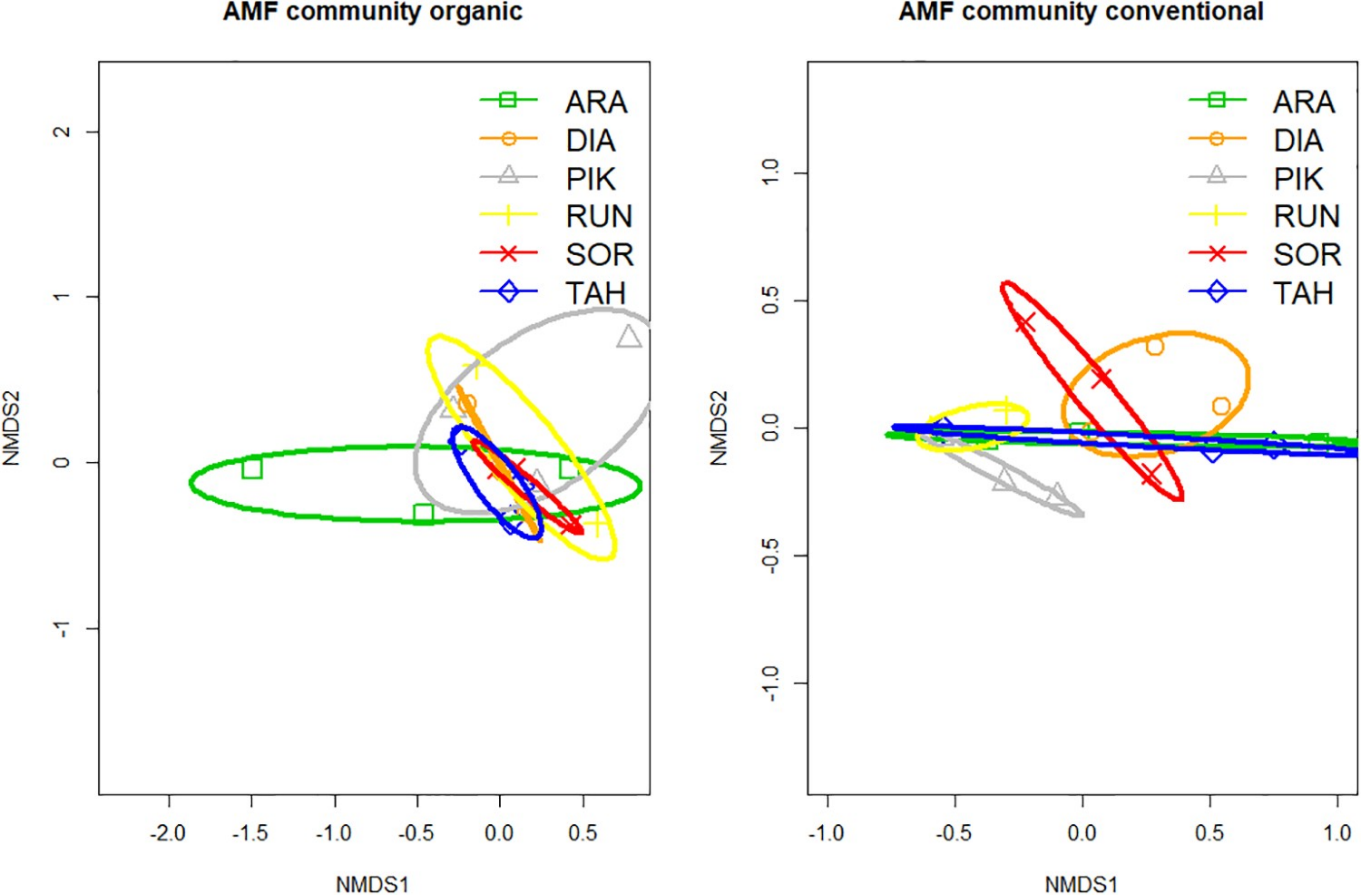
Nonmetric multidimensional scaling (NMDS) of AM fungal communities in roots of wheat cultivars grown with organic (left) and conventional (right) field soil inoculum. ARA: Arabella 2012, DIA: Diamant: 1929, PIK: Pikker 1959, RUN: Runar 1972, SOR: Sorbas 2016, and TAH: Tähti 1972. Each point represents one sample; ellipses show one standard error around group centroids.

AM fungal communities in the roots of wheat plants differed significantly when grown in the organic or conventional field soils (PERMANOVA R^2^ = 0.23; p < 0.05). While AM fungal richness and read abundance was not significantly affected by soil origin across all cultivars, there was a significant positive effect of cultivar age on AM fungal taxon composition (F_1,33_ = 6.66, P = 0.02). The two cultivars that exhibited the greatest difference in measured plant traits when grown with either organic or conventional field soils, Pikker and Arabella, exhibited a divergence of AM fungal community composition when grown in different soils (Fig 5), but did not differ significantly in the number of AM fungal taxa present nor their read abundance.

## Discussion

The overall effects of AM fungal inoculum originating from organically and conventionally managed fields on the growth and yield of wheat did not differ greatly. However, some spring wheat cultivars differed from each other significantly, where Diamant (year of release: 1929) exhibiting the lowest yield and Arabella (year of release: 2012) the highest yield under the given soil conditions. They also exhibited varying inoculation responses, showing the differences of the effects of organic and conventional inocula, with that of Pikker (1959) the most positive and that of Arabella (2012) the most negative. The use of organic field AM fungal inoculum resulted in slightly taller plant individuals. However, the differentiating effect of organic and conventional field inoculum was evident only in two out of the six wheat cultivars. Pikker showed relatively higher yield and better growth in the presence of organic field inoculum, while Arabella exhibited relatively lower yield and growth when the organic field inoculum was used. These varieties harboured different AM fungal communities in their roots as well.

During recent years there have been developments in the so-called integrated mycorrhizal technology, i.e. harnessing the mycorrhizal symbiosis for sustainable intensification in agriculture [2,52,53]. At the same time, minimal chemical input may encourage mycorrhiza—crop associations, but it often incurs yield-reducing trade-offs [22]. Although AM fungal communities in organic and conventional soils differed, our experiment did not bring forth a general unidirectional effect of organic field inoculum on wheat growth and yield, which is consistent with earlier studies [27].

Despite evidence showing a positive effect of mycorrhizal fungal inoculation, some earlier studies have shown that inoculation with AM fungi may reduce growth and yield of wheat, in comparison with non-mycorrhizal controls [11,12,54]. The overall effect observed in our study was largely consistent with a reduction in growth and yield. Our results indicated differential responses of cultivars to organic compared to conventional inoculation. While cultivar Pikker exhibited an overall positive inoculation response, the overall effect on cultivar Arabella was negative. At the same time, Arabella responded positively to conventional inoculum (S1 Fig). This study did not find an appealing association among fungal identities, inoculum type and cultivars (S2 Table). Thus, further studies have to disentangle the taxon-specific effects of AM fungi on particular wheat cultivars.

Comparing the effects of organic and conventional AM fungal field inoculum on crop growth and yield can importantly inform efforts to increase the effectiveness of sustainable agriculture. It has been known that cultivars of wheat exhibit different responses to mycorrhizal fungal inoculation [11,12,19,20]. Although earlier studies found that older cultivars strongly responded to AM fungal inoculation, later studies provide mixed results [16,18,21]. This study found some support for older cultivar responding differently than recent cultivars. Specifically, one of the oldest cultivars, Pikker, responded more positively to organic field inoculation than other cultivars, while one of the most recent cultivars, Arabella, responded more positively to conventional field inoculation than other cultivars.

Interestingly, the positively responding to organic field inoculum cultivar Pikker was the only cultivar in the experiment that was developed in Estonia (in 1959). It is one of the tallest, oldest, least agrochemically-demanding and traditionally cropped cultivars in Estonia. One may hypothesize that the positive response of Pikker to organic field inoculation may be related to its adaptation to locally occurring AM fungal communities. Although some AM fungi are relatively efficient dispersers, there remain regional differences in the composition of AM fungal communities [55]. Osborne et al. [56] proposed that AM fungi may promote the divergent adaptation of natural plant species. Similarly, enhancement of a locally bred cultivar by local AM fungal communities may reflect an adaptation of the cultivar to the locally occurring AM fungal communities [57]. This working hypothesis should be tested in further observational and experimental studies. At the same time, growth of cultivar Arabella was increased by conventional field-originating inoculation. This cultivar originated in Poland and is known to grow well under modern intensive agrochemical agriculture conditions.

Thirkell, et al. [22] warned that AM fungal colonization does not necessarily translate directly into enhanced plant performance or crop yield. However, even in the absence of easily measurable plant performance effect, management of soil AM fungi can benefit crop nutrient uptake [20]. Furthermore, the effects of AM fungal inoculation on ecosystem services other than crop production, such as the reduction of nutrient loss, erosion or pathogen damage, are also worthy for future study [58]. A comprehensive understanding of the ecological roles of AM fungi in agricultural systems will help us to move closer to integrated mycorrhizal technology [52] and feed a growing human population.

## Supporting information

S1 Fig Comparison between average Inoculation Responsiveness (IR) and Mycorrhizal Responsiveness (MR) of wheat cultivar traits: (a) plant height, (b) root weight, (c) shoot weight, (d) grain weight, (e) proportional grain weight (for short, grain ratio), and (f) root colonization. DIA: Diamant: 1929, PIK: Pikker 1959, TAH: Tähti 1972, RUN: Runar 1972, ARA: Arabella 2012, SOR: Sorbas 2016. Differences among cultivars could not be statistically tested due to the fact that this proxy was based on average values (i.e. there was a lack of within-cultivar variability per plant trait). Black circles show Inoculation Responsiveness (IR) on standardized data as ((average plant trait value growing with organic field inoculum—average plant trait value growing with conventional field inoculum). Green triangles show mycorrhizal responsiveness (MR) on standardized data as ((average plant trait value growing with organic field inoculum—average plant trait value growing with sterile soil). Brown crosses show mycorrhizal responsiveness (MR) on standardized data as ((average plant trait value growing with conventional field inoculum—average plant trait value growing with sterile soil).
(TIFF)

S2 Fig AM fungal taxon accumulation curves in relation to sequencing depth per sample for organic and conventional field soil inoculation treatments. Taxon accumulation curves showed no relationship between the number of sequences obtained from a sample and the AM fungal richness of that sample.
(PNG)

S1 Table Summary of the data based on what root colonization was calculated. DIA: Diamant: 1929, PIK: Pikker 1959, TAH: Tähti 1972, RUN: Runar 1972, ARA: Arabella 2012, SOR: Sorbas 2016.
(DOCX)

S2 Table Number of sequences of arbuscular mycorrhizal fungi per taxon identity, inoculum type and cultivar. DIA: Diamant: 1929, PIK: Pikker 1959, TAH: Tähti 1972, RUN: Runar 1972, ARA: Arabella 2012, SOR: Sorbas 2016.
(DOCX)

S1 File (CSV)
