# Supplementary figures and images for: Different wheat cultivars exhibit variable responses to inoculation with arbuscular mycorrhizal fungi from organic and conventional farms
Source: PLoS One. 2020 May 29;15(5):e0233878. doi: 10.1371/journal.pone.0233878 (PMC7259642; doi:10.1371/journal.pone.0233878)

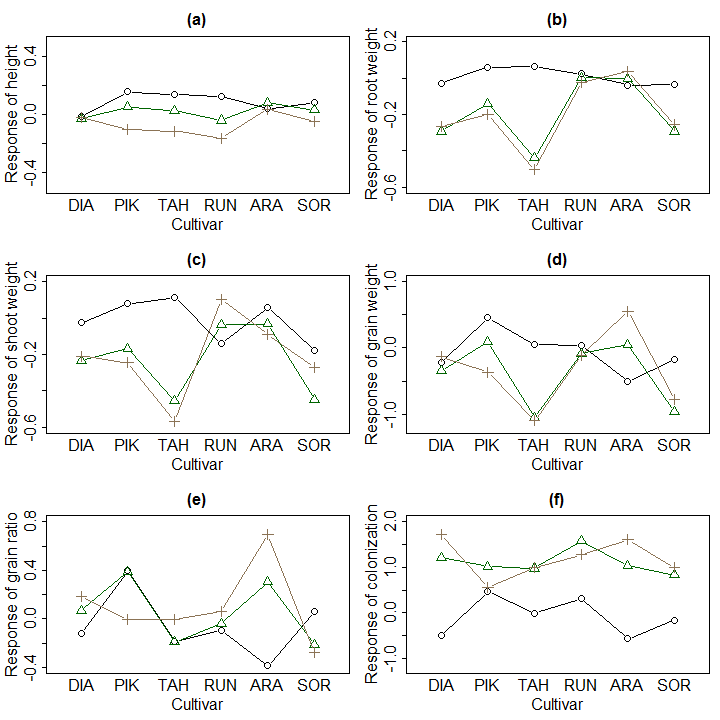

Supplement: S1 Fig — DIA: Diamant: 1929, PIK: Pikker 1959, TAH: Tähti 1972, RUN: Runar 1972, ARA: Arabella 2012, SOR: Sorbas 2016. Differences among cultivars could not be statistically tested due to the fact that this proxy was based on average values (i.e. there was a lack of within-cultivar variability per plant trait). Black circles show Inoculation Responsiveness (IR) on standardized data as ((average plant trait value growing with organic field inoculum—average plant trait value growing with conventional field inoculum). Green triangles show mycorrhizal responsiveness (MR) on standardized data as ((average plant trait value growing with organic field inoculum—average plant trait value growing with sterile soil). Brown crosses show mycorrhizal responsiveness (MR) on standardized data as ((average plant trait value growing with conventional field inoculum—average plant trait value growing with sterile soil). (TIFF) [file pone.0233878.s001.tiff]

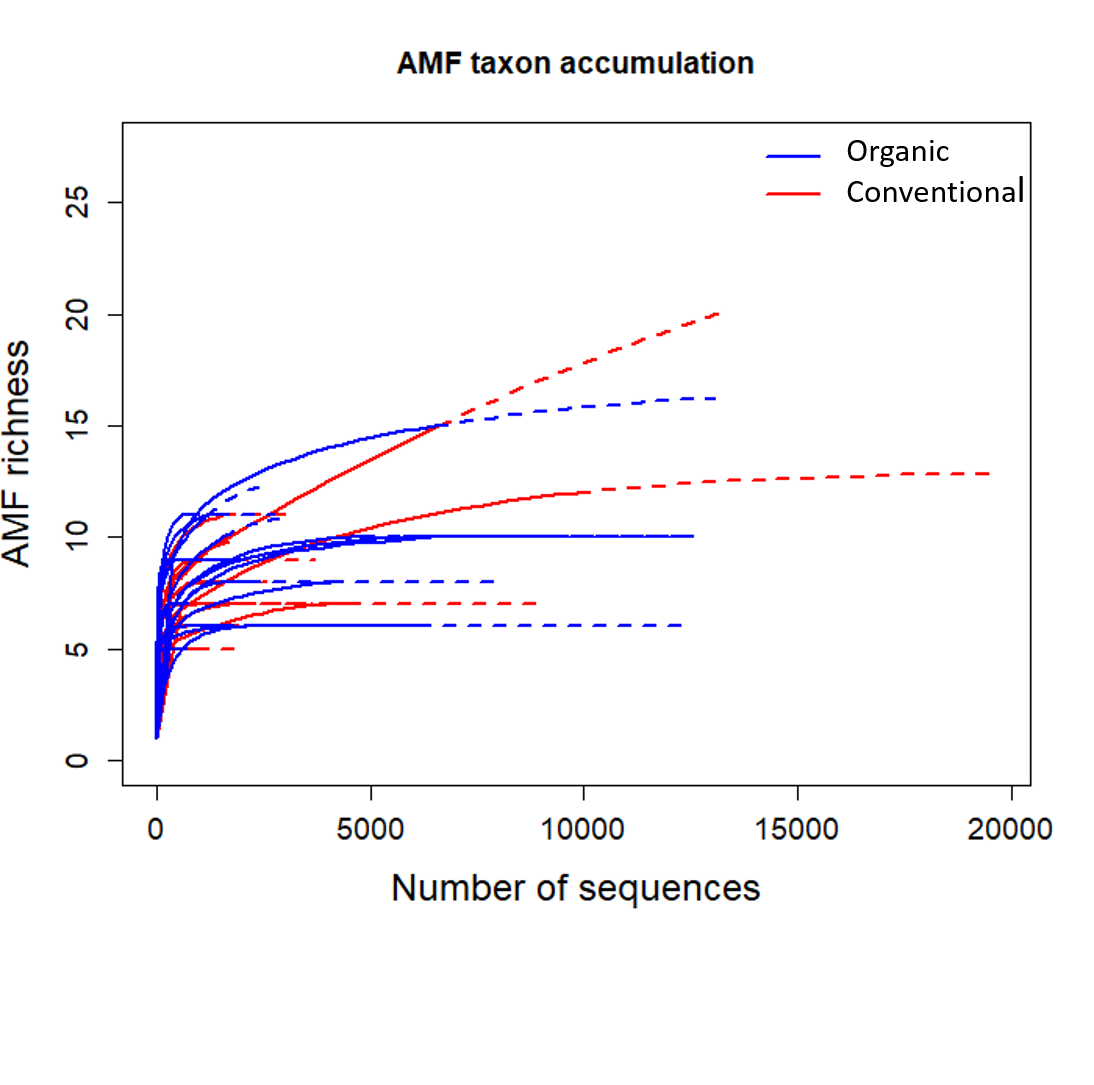

Supplement: S2 Fig — Taxon accumulation curves showed no relationship between the number of sequences obtained from a sample and the AM fungal richness of that sample. (PNG) [file pone.0233878.s002.png]
