# Supplementary material for: Different wheat cultivars exhibit variable responses to inoculation with arbuscular mycorrhizal fungi from organic and conventional farms
Source: PLoS One. 2020 May 29;15(5):e0233878. doi: 10.1371/journal.pone.0233878 (PMC7259642; doi:10.1371/journal.pone.0233878)
Supplement: S1 Table — DIA: Diamant: 1929, PIK: Pikker 1959, TAH: Tähti 1972, RUN: Runar 1972, ARA: Arabella 2012, SOR: Sorbas 2016. (DOCX) [file pone.0233878.s003.docx]

**Table S1.** Summary of the data based on what root colonization was calculated. DIA: Diamant: 1929, PIK: Pikker 1959, TAH: Tähti 1972, RUN: Runar 1972, ARA: Arabella 2012, SOR: Sorbas 2016.

|  | Conventional | | | | | | Organic | | | | | |
| --- | --- | --- | --- | --- | --- | --- | --- | --- | --- | --- | --- | --- |
| Cultivar | DIA | PIK | TAH | RUN | ARA | SOR | DIA | PIK | TAH | RUN | ARA | SOR |
| Hyphae | 13±4 | 5±1 | 0±0 | 14±0 | 7±3 | 2±0 | 11±8 | 20±0 | 9±4 | 14±16 | 4±5 | 8±6 |
| Vesicle | 3±1 | 1±0 | 1±0 | 2±0 | 2±1 | 0±0 | 2±0 | 2±1 | 3±1 | 3±4 | 2±0 | 2±1 |
| Arbuscule | 1±0 | 2±0 | 0±0 | 2±0 | 1±0 | 0±0 | 3±3 | 3±0 | 4±0 | 13±11 | 4±2 | 7±0 |
| Dark septate hyphae | 30±6 | 11±7 | 25±0 | 16±0 | 34±15 | 24±1 | 22±6 | 18±3 | 16±9 | 16±11 | 20±8 | 15±10 |
| Dark septate spores | 1±0 | 0±0 | 0±0 | 0±0 | 2±0 | 2±0 | 2±1 | 1±0 | 2±1 | 2±1 | 3±3 | 1±1 |
| Root colonization | 46±5 | 15±10 | 26±0 | 34±0 | 43±14 | 26±1 | 38±2 | 27±14 | 26±13 | 37±23 | 28±7 | 21±13 |
