# Supplementary material for: Different wheat cultivars exhibit variable responses to inoculation with arbuscular mycorrhizal fungi from organic and conventional farms
Source: PLoS One. 2020 May 29;15(5):e0233878. doi: 10.1371/journal.pone.0233878 (PMC7259642; doi:10.1371/journal.pone.0233878)
Supplement: S2 Table — DIA: Diamant: 1929, PIK: Pikker 1959, TAH: Tähti 1972, RUN: Runar 1972, ARA: Arabella 2012, SOR: Sorbas 2016. (DOCX) [file pone.0233878.s004.docx]

**Table S2** Number of sequences of arbuscular mycorrhizal fungi per taxon identity, inoculum type and cultivar. DIA: Diamant: 1929, PIK: Pikker 1959, TAH: Tähti 1972, RUN: Runar 1972, ARA: Arabella 2012, SOR: Sorbas 2016.

|  |  |  |  | Conventional inoculum | | | | | | | Organic inoculum | | | | | | |
| --- | --- | --- | --- | --- | --- | --- | --- | --- | --- | --- | --- | --- | --- | --- | --- | --- | --- |
| **Virtual taxon** | **Family** | **Genus** | **Species** | DIA | PIK | TAH | RUN | ARA | SOR | **Total** | DIA | PIK | TAH | RUN | ARA | SOR | **Total** |
| VT388 | Glomeraceae | *Glomus* | *Sp* | 3412 | 750 | 3443 | 2303 | 6502 | 360 | 16770 | 1927 | 5866 | 1601 | 4525 | 753 | 2357 | 17029 |
| VT113 | Glomeraceae | *Rhizoglomus* | *irregulare* | 2222 | 1965 | 5654 | 7577 | 3030 | 235 | 20683 | 626 | 1290 | 365 | 2200 | 65 | 301 | 4847 |
| VT115 | Glomeraceae | *Rhizoglomus* | *irregulare* | 2327 | 1120 | 3815 | 5091 | 2308 | 185 | 14846 | 515 | 772 | 282 | 1214 | 47 | 260 | 3090 |
| VT67 | Glomeraceae | *Funneliformis* | *coronatum* | 57 | 273 | 214 | 57 | 0 | 35 | 636 | 163 | 24 | 386 | 29 | 0 | 38 | 640 |
| VT114 | Glomeraceae | *Rhizoglomus* | *irregulare* | 38 | 52 | 94 | 119 | 132 | 4 | 439 | 107 | 1471 | 49 | 1031 | 227 | 31 | 2916 |
| VT105 | Glomeraceae | *Rhizoglomus* | *intraradices* | 94 | 25 | 172 | 77 | 202 | 20 | 590 | 141 | 134 | 79 | 131 | 23 | 120 | 628 |
| VT444 | Paraglomeraceae | *Paraglomus* | *sp* | 112 | 53 | 18 | 32 | 28 | 78 | 321 | 20 | 29 | 43 | 78 | 29 | 70 | 269 |
| VT165 | Glomeraceae | *Glomus* | *sp* | 1 | 0 | 0 | 2 | 0 | 0 | 3 | 244 | 16 | 175 | 0 | 4 | 88 | 527 |
| VT57 | Claroideoglomeraceae | *Claroideoglomus* | *sp* | 0 | 0 | 4 | 4 | 0 | 0 | 8 | 0 | 453 | 3 | 0 | 3 | 0 | 459 |
| VT281 | Paraglomeraceae | *Paraglomus* | *laccatum* | 12 | 2 | 67 | 4 | 2 | 0 | 87 | 11 | 0 | 0 | 2 | 0 | 0 | 13 |
| VT340 | Claroideoglomeraceae | *Claroideoglomus* | *sp* | 1 | 0 | 0 | 2 | 0 | 0 | 3 | 0 | 54 | 0 | 0 | 0 | 0 | 54 |
| VT60 | Diversisporaceae | *Diversispora* | *sp* | 44 | 0 | 0 | 0 | 0 | 0 | 44 | 0 | 0 | 0 | 0 | 0 | 0 | 0 |
| VT245 | Archaeosporaceae | *Archaeospora* | *trappei* | 0 | 0 | 0 | 0 | 0 | 0 | 0 | 9 | 1 | 2 | 0 | 10 | 14 | 36 |
| VT214 | Glomeraceae | *Glomus* | *sp* | 0 | 0 | 0 | 0 | 0 | 0 | 0 | 0 | 29 | 7 | 0 | 0 | 0 | 36 |
| VT65 | Glomeraceae | *Funneliformis* | *caledonium* | 0 | 3 | 0 | 1 | 0 | 0 | 4 | 0 | 0 | 13 | 0 | 0 | 0 | 13 |
| VT143 | Glomeraceae | *Glomus* | *sp* | 1 | 5 | 6 | 0 | 0 | 0 | 12 | 0 | 3 | 1 | 6 | 0 | 3 | 13 |
| VT188 | Glomeraceae | *Glomus* | *sp* | 0 | 0 | 0 | 0 | 0 | 0 | 0 | 7 | 0 | 2 | 0 | 0 | 5 | 14 |
| VT387 | Glomeraceae | *Glomus* | *sp* | 1 | 3 | 0 | 4 | 0 | 0 | 8 | 0 | 0 | 0 | 0 | 0 | 0 | 0 |
| VT56 | Claroideoglomeraceae | *Claroideoglomus* | *sp* | 0 | 0 | 0 | 0 | 0 | 0 | 0 | 0 | 0 | 0 | 0 | 6 | 0 | 6 |
| VT295 | Glomeraceae | *Glomus* | *sp* | 2 | 0 | 1 | 1 | 0 | 0 | 4 | 0 | 0 | 0 | 0 | 0 | 0 | 0 |
| VT402 | Claroideoglomeraceae | *Claroideoglomus* | *sp* | 1 | 0 | 0 | 0 | 0 | 0 | 1 | 0 | 2 | 1 | 0 | 0 | 0 | 3 |
| VT435 | Paraglomeraceae | *Paraglomus* | *sp* | 0 | 0 | 1 | 0 | 0 | 0 | 1 | 0 | 0 | 0 | 2 | 0 | 0 | 2 |
| VT361 | Glomeraceae | *Glomus* | *sp* | 1 | 0 | 0 | 0 | 0 | 0 | 1 | 0 | 0 | 0 | 0 | 0 | 1 | 1 |
| VT270 | Glomeraceae | *Glomus* | *sp* | 0 | 0 | 0 | 0 | 0 | 0 | 0 | 1 | 1 | 0 | 0 | 0 | 0 | 2 |
|  |  |  | *Sequences* | 8326 | 4252 | 13490 | 15274 | 12204 | 917 |  | 3773 | 10145 | 3009 | 9218 | 1167 | 3289 |  |
|  |  |  | *Richness* | 16 | 11 | 12 | 14 | 7 | 7 |  | 12 | 15 | 15 | 10 | 10 | 12 |  |
